# Cigarette smoke destabilizes NLRP3 protein by promoting its ubiquitination

**DOI:** 10.1186/s12931-016-0485-6

**Published:** 2017-01-05

**Authors:** SeungHye Han, Jacob A. Jerome, Alyssa D. Gregory, Rama K. Mallampalli

**Affiliations:** 1 Department of Medicine, The Acute Lung Injury Center of Excellence, University of Pittsburgh, Pittsburgh, PA USA; 2 Department of Medicine, Division of Pulmonary, Allergy, and Critical Care Medicine, University of Pittsburgh, 15213 Pittsburgh, PA USA; 3 Medical Specialty Service Line, Veterans Affairs Pittsburgh Healthcare System, Pittsburgh, PA USA

**Keywords:** NLRP3, Cigarette smoke, Ubiquitin

## Abstract

**Background:**

Cigarette smoke suppresses innate immunity, making smokers more susceptible to infection. The NLRP3 inflammasome is a multi-protein complex that releases interleukin (IL) -1β and IL -18. These cytokines are critical for a timely host response to pathogens. Whether cigarette smoke affects NLRP3 protein levels, and its ability to form an inflammasome, is not known.

**Methods and results:**

Using the human monocyte THP1 cell line and C57BL/6 mice, we show that cigarette smoke decreases NLRP3 levels in cells by increasing ubiquitin-mediated proteasomal processing. Half-life of NLRP3 is shortened with the exposure to cigarette smoke extract. Cigarette smoke extract reduces cellular NLRP3 protein abundance in the presence of lipopolysaccharide, a known inducer of NLRP3 protein, thereby decreasing the formation of NLRP3 inflammasomes. The release of IL-1β and IL-18 by inflammasome activation is also decreased with the exposure to cigarette smoke extract both in THP1 cells and primary human peripheral blood macrophages.

**Conclusions:**

Cigarette smoke extract decreased NLRP3 protein abundance via increased ubiquitin-mediated proteasomal processing. The release of IL-1β and IL-18 is also decreased with cigarette smoke extract. Our findings may provide mechanistic insights on immunosuppression in smokers and unique opportunities to develop a strategy to modulate immune function.

## Background

Nucleotide-binding oligomerization (NOD) -like receptors (NLRs) are cytosolic pattern recognition receptors responsible for detecting pathogen- or danger-associated molecular patterns (PAMPs or DAMPs), and are a critical surveillance system for innate immunity. Most NLRs share common structural characteristics: a C-terminal leucine-rich repeat domain that recognizes PAMPs or DAMPs, a central NOD domain, and a variable N-terminal effector domain [1]. They are categorized into five families based on their N-terminal domains. NLRP3 (NALP3) has a pyrin N-terminal domain which binds with the adaptor protein, ASC, to recruit pro-caspase-1 (p45), forming a multi-protein complex termed the inflammasome. Upon inflammasome activation, active caspase-1 (p20 or p10) is cleaved and pro-inflammatory cytokines such as interleukin (IL) -1β and -18, which play an important role in host defense against infection, are subsequently released.

Cigarette smoking has been known to increase susceptibility to infection likely from dysregulation of immune function [2], but the precise underlying molecular mechanisms remain unclear. A previous study showed that cigarette smoke alone does not induce secretion of IL-1β, an inflammasome cytokine, in human monocyte THP1 cells [3]. On the other hand, NLRP3 appears to be required for bronchoalveolar secretion of IL-1β in response to cigarette smoke in an in vivo murine model [4]. However, it is not known whether cigarette smoke affects NLRP3 cellular concentrations or how it interacts with other pathogens to affect cellular protein levels. This question is important, as the identification of an effect, and underlying mechanism, could provide us a therapeutic target to control dysregulated immune function in smokers.

The aim of our study was to investigate the molecular basis for effects of cigarette smoke extract (CSE) on the NLRP3 inflammasome, a component also activated by lipopolysaccharide (LPS). We investigated if CSE interacts with LPS and modulates NLRP3 inflammasome activity and cytokine release. To this end, we utilized human monocyte THP1 cells and primary human peripheral blood macrophages, and C57BL/6 mice to assess the in vivo effects of cigarette smoke.

## Methods

### Antibodies and reagents

Antibodies against ubiquitin and IL-1β were obtained from Cell Signaling Technology (Danvers, MA). Leupeptin, ATP, and antibodies against β-actin and GAPDH were acquired from Sigma-Aldrich (St. Louis, MO). NLRP6 antibodies were purchased from Abcam (Cambridge, MA). NLRP3 antibodies were from Adipogen (San Diego, CA). Antibody against ASC was obtained from Santa Cruz Biotechnology (Santa Cruz, CA). Protein A/G agarose beads, pcDNA3.1D TOPO cloning kits, LPS, and antibodies targeting the V5 tag were purchased from Thermo Fisher Scientific (Waltham, MA). IL-18 antibodies were acquired from MBL International (Woburn, MA). Caspase-1 antibodies were obtained from R&D Systems (Minneapolis, MN). Cycloheximide (CHX) was purchased from Enzo Life Sciences (Farmingdale, NY). MG-132 was purchased from Ubiquitin-Proteasome Biotechnologies (Aurora, CO). CSE in a vacuum sealed bottle was purchased from Murty Pharmaceuticals (Lexington, KY).

### Cell culture

Human monocyte THP1 cells were purchased from Sigma-Aldrich. Human peripheral blood macrophages and human macrophage cell culture medium were purchased from Celprogen (Torrance, CA). The clonal primary macrophages were derived from human peripheral blood, and confirmed positive for Mcl-1, CD4, CD14, CD206, CD11b/CR3, CD2, and CD19 expression per the company. RPMI 1640 medium was purchased from Thermo Fisher Scientific. Fetal bovine serum (FBS) was purchased from Gemini (Sacramento, CA). THP1 cells were cultured in RPMI 1640 medium supplemented with 10% FBS. Human peripheral blood macrophages were cultured in human macrophage cell culture medium supplemented with 10% FBS. For the half-life experiments, CHX was used at a concentration of 40 μg/mL in fresh medium without FBS supplement, avoiding the possible breakdown of CHX when it is mixed with an alkaline substance (i.e., nicotine from CSE). For analysis of secreted proteins, the medium was removed from treated cells with the same total cell number and medium volume per well and precipitated with trichloroacetic acid (TCA). The precipitated pellet was then mixed with sodium dodecyl sulfate–polyacrylamide gel electrophoresis (SDS-PAGE) sample loading buffer and analyzed by immunoblotting.

### qRT-PCR

RNA was isolated from THP1 cells using the RNeasy Mini Kit from Qiagen (Valencia, CA) per the protocol supplied in the kit. The concentration of each RNA sample was measured, followed by conversion to cDNA using the High-Capacity RNA-to-cDNA kit from Thermo Fisher Scientific. Real-time PCR was carried out in a C1000 Thermal Cycler from Bio-Rad (Hercules, CA) using SYBR Select Master Mix from Thermo Fisher Scientific per the included protocol. The primers used were *NLRP3* (5′-ATGAGTGCTGCTTCGACATC-3′, 5′-TTGTCACTCAGGTCCAGCTC-3′), and *GAPDH* (5′-ATCATCCCTGCCTCTACTGC-3′, 5′-GTCAGGTCCACCACTGACAC-3′).

### Immunoprecipitation and immunoblotting

Cells were collected in lysis buffer (0.25% Triton X-100 in PBS and 1:1000 protease inhibitor mixture) and sonicated for 12 s, followed by centrifugation at 16,100 × g for 10 min. The cell lysate was then incubated and rotated with 5 μL of anti-ubiquitin antibody at room temperature for 1 h. Each sample was then incubated with 30 μL of protein A/G agarose beads and rotated overnight at 4 °C. After incubation overnight, the beads were spun down at 0.1 × g for 3 min and then washed with lysis buffer a total of 3 times. SDS-PAGE sample loading buffer was added to the beads and they were boiled for 5 min before immunoblot analysis. Immunoblotting was carried out as follows; Equal amounts of protein in sample loading buffer were separated by gel electrophoresis, and transferred onto nitrocellulose membranes [5]. Restore PLUS Western Blot Stripping Buffer from Thermo Fisher Scientific (Waltham, MA) was used to reprobe membranes to detect multiple proteins.

### Cloning and mutagenesis

Human *NLRP3* cDNA was cloned into a pcDNA3.1D/V5-His vector provided in the pcDNA3.1D TOPO cloning kit. Site directed mutagenesis of *NLRP3* was performed using the QuikChange II XL kit from Agilent Technologies (Santa Clara, CA) as previously described [6].

### Transfection

2.5 × 10^6^ Human peripheral blood macrophages were suspended in 100 μL of 20 mM HEPES in PBS and were mixed with 4 μg of either WT *NLRP3*-V5 or K689R *NLRP3*-V5 plasmid DNA in a cuvette. The cells were nucleofected using the Y-010 protocol on an Amaxa Nucleofector II machine (Basel, Switzerland). After transfection, 1 mL of 10% FBS human macrophage cell culture medium was added to each cuvette. The samples were then transferred to 6-well plates containing 1 mL of 10% FBS human macrophage cell culture medium, for a total of 2 mL of culture medium. The cells were grown until they reached approximately 50% confluency (~48–72 h) before half-life experiments were initiated.

### Animal study

Male C57BL/6 mice ranging from 8 to 12 weeks of age were purchased from the Jackson Laboratory (Bar Harbor, ME) and exposed to 4 non-filtered cigarettes (University of Kentucky research cigarettes, Lot number 1R5F), 5 days per week, for a total of 6 months. The mice were deposited in a smoking chamber which allows the restrained mice to get direct cigarette smoke exposure towards their nose [7]. A mouse was exposed to 8.32 mg total particulate matter per day via the targeted delivery system [8]. Age-matched littermates were used as controls and were exposed to filtered air. After 6 months, the mice were sacrificed by administration of CO_2_. Following euthanasia, the lungs were immediately extracted and frozen in liquid nitrogen for storage at -80 °C. The lungs were then homogenized in lysis buffer (1% Triton X-100 in PBS and 1:1000 protease inhibitor mixture), before analysis via SDS-PAGE and immunoblotting. The protocol described was approved by the University of Pittsburgh Institutional Animal Care and Use Committee (Protocol #: 12101008).

### Statistical analysis

A Mann-Whitney *U* test or a Kruskal-Wallis equality of populations rank test were used to compare experimental groups. We employed non-parametric methods as our sample sizes were relatively small to check a normal sample distribution. All analyses were performed two-tailed, using Stata Statistical Software: Release 13.0 (StataCorp. 2013. College Station, TX: StataCorp LP).

## Results

### Cigarette Smoke Extract (CSE) decreases NLRP3 protein

**Fig. 1:**
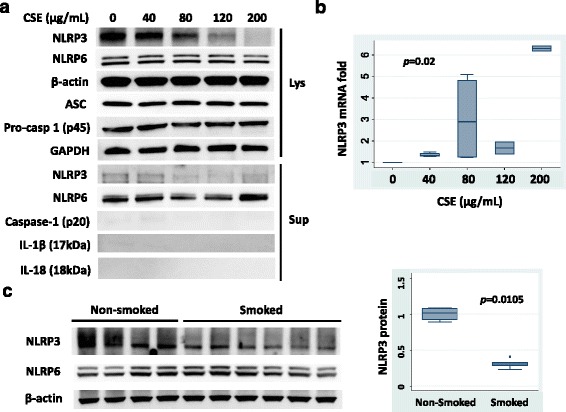
Cigarette Smoke Extract (CSE) decreases NLRP3 abundance. **a** THP1 cells (total 3 × 10^6^ cells) were treated with the indicated concentrations of CSE for 16 h. Cell lysates (Lys) and culture medium (supernatants, Sup) were collected to measure protein levels of NLRP3, NLRP6 (negative control), ASC, Caspase-1, IL-1β, IL-18 and β-actin/GAPDH (loading control) by immunoblotting. Membranes were stripped to detect multiple different proteins. Immunoblot shown is representative of four independent experiments. The lysate blots are from two blots that are loaded at the same time from identical cell lysates. NLRP3, NLRP6 and β-actin are obtained from one, and p45, ASC, and GAPDH are from another blot. For supernatant blots, NLRP3, NLRP6, and p20 are from the same blot, and IL-1β and IL-18 are from a different blot. **b** THP1 cells were treated with the indicated concentrations of CSE for 16 h before RNA isolation. Shown is the *NLRP3* mRNA expression fold changes determined by qRT-PCR in a box plot. Data are representative of four independent experiments. *P* value was determined by a Kruskal-Wallis test. **c** Whole lung lysates from non-smoked or smoked C57BL/6 mice were immunoblotted for NLRP3, NLRP6, and β-actin protein. The relative densitometries of NLRP3 protein for the immunoblots are shown in the right panel. *P* value was determined by a Mann-Whitney test

NLRP3 protein levels selectively decreased after CSE exposure in a dose-dependent fashion in human monocyte THP1 cells (Fig. 1a). We chose 16 h for the duration of CSE exposure as the effect was maximized after 12–16 h (data not shown). Other components of the inflammasome such as pro-caspase-1 (p45) and ASC were not changed after CSE exposure. It has been reported that oligomeric NLRP3 inflammasome particles are released from macrophages after activation of the inflammasome [9]. To exclude the possibility that cellular levels of NLRP3 protein decreased due to the secretion of NLRP3 protein as extracellular oligomeric complexes, we measured the abundance of secreted proteins in the culture medium (supernatant) by immunoblotting. Minimal levels of NLRP3 protein were detected without significant changes after CSE exposure (Fig. 1a). Neither active caspase-1 (p20) nor cytokines such as IL-1β and IL-18 were detected in supernatants, which is consistent with prior studies [3]. Cell viability was not significantly different with CSE exposure (mean viability of 89% ± standard deviation (S.D.) of 6%, *p* = 0.30 by Kruskal-Wallis test). The steady-state mRNA expression of *NLRP3*, however, tended to increase after CSE exposure (Fig. 1b). This suggests that the CSE-induced change in protein levels is mainly mediated by post-translational regulation, and the increase in mRNA expression of *NLRP3* is likely a compensatory response to the decreased NLRP3 protein levels. In order to assess in vivo effects of CSE, we measured NLRP3 protein levels in mouse lung lysates. The amount of NLRP3 protein was also selectively reduced in lung tissue from mice that were exposed to cigarette smoke for 6 months, compared with those from non-smoking control mice (Fig. 1c).

### CSE shortens NLRP3 half-life via increased ubiquitin-mediated proteasomal degradation

**Fig. 2:**
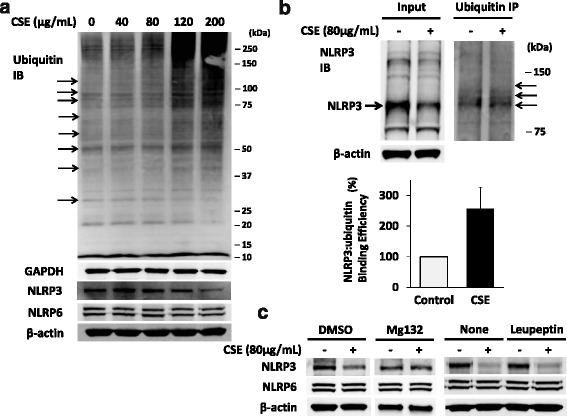
CSE triggers NLRP3 protein degradation via the ubiquitin proteasome system. **a** *Above*: Overall abundance of ubiquitin (*arrows*) increases with CSE exposure. THP1 cells were incubated with the indicated concentrations of CSE for 20 h. *Below*: Protein levels of ubiquitin, NLRP3, NLRP6, GAPDH, and β-actin were determined by immunoblotting. Membranes were stripped to detect multiple proteins. Ubiquitin and GAPDH are from the same blot, and NLRP3, NLRP6, and β-actin are from a separate blot. **b** THP1 cells were incubated with or without CSE at 80 μg/mL for 17 h. Cells were lysed and immunoblotted for NLRP3 protein (*left panel*, input; cell lysates). In the right panel, ubiquitin was immunoprecipitated, followed by NLRP3 immunoblotting. Modestly increased intensity of several bands (*arrows*) with CSE exposure are observed, and corrected for input the results are shown graphically (*bottom*). The bar graph represents mean ± S.D. from two independent experiments. **c** THP1 cells were incubated with or without CSE at 80 μg/mL, and with or without MG-132 at 20 μM or leupeptin at 40 μM for 16 h. As MG-132 is water insoluble, dimethyl sulfoxide (DMSO) was used as a vehicle. Control was normalized by the amount of DMSO used (0.1%). Immunoblotting was performed to determine NLRP3, NLRP6, and β-actin protein levels. The blots shown are representative of two to three independent experiments

Overall cellular ubiquitination was increased in cells with CSE exposure in a dose-dependent manner (Fig. 2a). We have previously showed that NLRP3 protein is degraded via the ubiquitin proteasome system [6]. To determine whether CSE increases NLRP3 ubiquitination, we measured the interaction between NLRP3 protein and ubiquitin by co-immunoprecipitation both at baseline and after CSE exposure. The NLRP3-ubiquitin interaction was increased by more than 100% after CSE exposure when densitometrically controlled for input loading (Fig. 2b). CSE induced degradation of NLRP3 protein was inhibited by MG132, a proteasome inhibitor, but not by a lysosomal inhibitor, leupeptin, confirming that the effect of CSE on NLRP3 protein occurs through the ubiquitin proteasome system (Fig. 2c). The NLRP3 protein mass was reduced by 55% ± 27% with CSE exposure, and the CSE-induced reduction was not changed significantly in the presence of DMSO (54% reduction ± S.D. of 41%, *p* = 0.77 by Mann-Whitney *U* test).

**Fig. 3:**
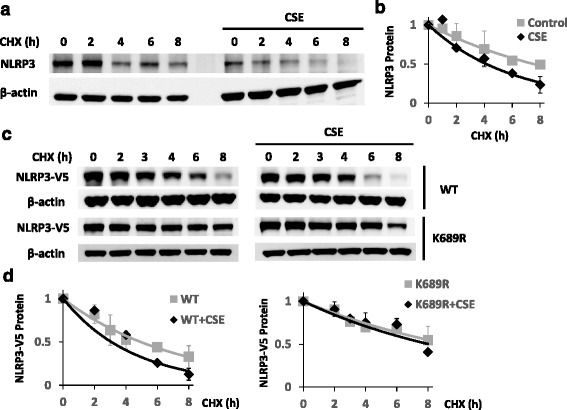
CSE decreases the half-life of the NLRP3 protein. **a** THP1 cells were incubated with or without CSE at 80 μg/mL for 18 h prior to CHX exposure at 40 μg/mL for the indicated time periods. Cells were collected and assayed for NLRP3 and β-actin (loading control) by immunoblotting for a half-life study. Representative images are shown. **b** Densitometric plots of NLRP3 protein decay versus time of CHX exposure with best fit lines, depicting the pooled data mean ± S.D. of three independent experiments. **c** Primary human macrophages from peripheral blood were transfected with 4 μg of either WT *NLRP3*-V5 or the point mutant K689R *NLRP3*-V5 plasmid. Following transfection, the cells were incubated with or without CSE at 120 μg/mL for 21 h, prior to CHX exposure at 40 μg/mL at different time points for a half-life study. Cells were collected and assayed for NLRP3-V5 and β-actin by immunoblotting. **d** Densitometric plots of adjusted NLRP3 protein decay over time under different conditions were best fitted. The half-life of WT NLRP3 protein was reduced with CSE exposure, while a K689R NLRP3 mutant was not. Data are mean ± S.D. of two independent experiments

Using cycloheximide to inhibit protein synthesis, the half-life of NLRP3 was also shortened after CSE exposure (Fig. 3a–b). We previously demonstrated that Lys-689 is a NLRP3 ubiquitination acceptor site [6]. We transfected primary human peripheral blood macrophages with wild-type and K689R *NLRP3* mutant plasmids, and measured the half-life of ectopically expressed NLRP3 protein with or without CSE exposure. The half-life of K689R NLRP3 mutant was not changed after CSE exposure, while the wild-type variant had a shortened half-life similar to the endogenous NLRP3 protein (Fig. 3c–d). These results suggest that CSE triggers ubiquitination at the K689 site to accelerate NLRP3 degradation because the K689R mutant exhibited a longer t ½. In both THP1 cells and primary human peripheral blood macrophages, the half-life of NLRP3 was slightly longer (~ 5–6 h in an unchallenged condition) than in U937 cells (~ 4 h in an unchallenged condition) [6].

### CSE attenuates LPS-induced release of IL-1β and IL-18

**Fig. 4:**
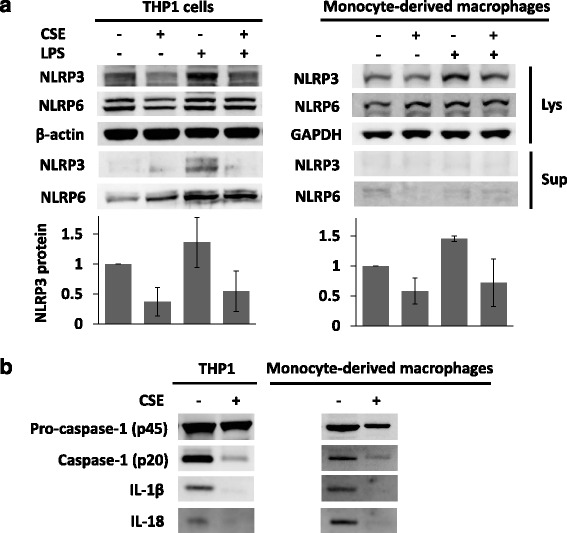
CSE reduces LPS-induced NLRP3 abundance and release of cytokines. **a** THP1 cells were incubated with or without 80 μg/mL of CSE, with or without 400 ng/mL of LPS, for 18 h (four groups; none, CSE, LPS, and LPS + CSE). Human primary macrophages derived from monocytes were incubated with or without 120 μg/mL of CSE, with or without 200 ng/mL of LPS, for 20 h. Protein levels of NLRP3, NLRP6, β-actin or GPADH were determined by immunoblotting. Culture medium (supernatants) were also collected for immunoblotting of NLRP3 and NLRP6. The relative densitometries of NLRP3 protein adjusted for loading control (β-actin or GPADH) are shown in the bottom panels. Data are mean ± S.D. of two independent experiments. **b** Equal numbers of THP1 (3 × 10^6^ cells) were plated in equivalent amounts of culture medium and incubated with or without 80 μg/mL of CSE, with or without 400 ng/mL of LPS, for 18 h. Cells were then exposed to 5 mM of ATP for 15 min. Culture medium was precipitated with TCA for immunoblotting of IL-1β, IL-18, and caspase-1. Also, equal numbers of human monocyte-derived macrophages in equal amounts of culture medium were incubated with or without 120 μg/mL of CSE, with or without LPS at 400 ng/mL, for 40 h. Cells were then exposed to 5 mM of ATP for 30 min. Cell lysates and culture medium were collected for immunoblotting. p45 and p20 are probed in the same blot, but presented in different exposure. IL-1β and IL-18 are from the same blot

We previously showed that LPS increases NLRP3 protein levels, thereby increasing the release of IL-1β and IL-18 in human inflammatory cells when inflammasomes are activated [6]. Thus, we examined whether CSE affects NLRP3 inflammasome activation and subsequent release of cytokines induced by LPS. As before, the amount of NLRP3 protein modestly increased with LPS both in THP1 cells and primary human monocyte-derived macrophages, and CSE alone produced a reduction in NLRP3 mass (Fig. 4a). Further, the potent effects of CSE on reduction of NLRP3 mass in cells were maintained despite addition of LPS to the culture medium (Fig. 4a). LPS prolongs the half-life of NLRP3 protein by reducing its degradation [6]. The combinatorial, yet opposite effects of LPS (inhibiting degradation) and CSE (promoting degradation) resulted in a 30–40% decrease in NLRP3 protein levels as shown in the lower panels of Fig. 4a. NLRP3 protein was not released into the culture medium after CSE exposure. CSE exposure of cells was sufficient to impair LPS-induced cleavage of active caspase-1, and thus the release of IL-1β and IL-18 both in THP1 cells and primary human monocyte-derived macrophages (Fig. 4b).

## Discussion

Our study reveals a unique observation that CSE decreases NLRP3 protein levels, mediated by increased ubiquitin proteasomal processing. Further, we demonstrate that CSE suppresses NLRP3 levels even in the presence of endotoxin thereby preventing the release of IL-1β and IL-18, critical cytokines for antimicrobial host defense. Our findings provide potential mechanistic insights for smoking-related immunosuppression, and the results may uncover unique opportunities to develop therapeutic strategies to modulate cytokine signaling. For example, small molecules that stabilize NLRP3 protein levels (e.g. targeting of NLRP3 deubiquitinating enzymes) might be one opportunity that emerges from the results of these and other studies.

Cigarette smoke has been known to dysregulate both innate and adaptive immune function, making smokers more susceptible to infection with worse outcomes [2, 10]. Specifically, smokers have increased susceptibility to bacterial pneumonia, tuberculosis, periodontitis and surgical infections [2]. The function of neutrophils and macrophages in smokers is defective, and they secrete lower levels of IL-6 and tumor necrosis factor (TNF) [11], which are crucial for early response to pathogens [12].

In addition, IL-1β and IL-18 are also known to play an important role in host defense. IL-1β activates the release of TNF and IL-6, and induces Th17 cell differentiation for cellular adaptive responses [13]. IL-18 is essential for the induction of IFN-γ and regulation of Th1 responses [14]. Both cytokines, IL-1β and IL-18, are synthesized as premature forms in cells, and cleaved by caspase-1 (p20 or p10) to be bioactive. Caspase-1 is activated by multi-protein complexes, inflammasomes, consisting of three components: a sensor NLR, adaptor ASC, and effector pro-caspase-1 (p45). The most studied is the NLRP3 inflammasome, which is associated with immune responses that limit microbial invasion, thereby protecting hosts [15]. A previous study showed that cigarette smoke decreases caspase-1 activity when THP1 cells are stimulated with asbestos [16]. However, it is not known whether cigarette smoke directly affects NLRP3 protein mass. Our study shows that CSE decreases the level of NLRP3 protein via increased degradation, most likely increased ubiquitin-mediated proteasomal processing. The CSE-induced degradation of NLRP3 was observed despite addition of LPS, a known inhibitor of NLRP3 ubiquitination that stabilizes the NLRP3 protein [6]. Release of IL-1β and IL-18 was also decreased after CSE exposure, likely from a decreased amount of activated NLRP3 inflammasome complex, as evidenced by reduced levels of active caspase-1 (Fig. 4b).

The ubiquitin proteasome system mediates disposal of the majority of proteins in cells. In lung epithelial cells, cigarette smoke increases total cellular poly-ubiquitinated proteins [17], which is consistent with our findings (Fig. 2a). CSE also induces the degradation of proteins involved with cell death and proliferation [18, 19]. Our studies indicate that CSE also suppresses immune function by modifying the activity of the ubiquitin proteasome system.

Previous studies suggest that the downstream products of NLRP3 inflammasomes such as IL-1β or IL-18 are associated with the pathophysiology of smoke-driven chronic obstructive pulmonary disease (COPD) although direct evidence to link NLRP3 protein with the disease is sparse. It is possible that the response to cigarette smoke could differ by cell type, model system, or kinetics. The level of IL-1β and/or IL-18 was increased in the lungs, lavage fluid, or sputum of COPD subjects or animals exposed to cigarette smoke [20–23]. However, cigarette smoke alone does not secrete IL-1β in THP1 cells [3], and we found that the release of IL-1β and IL-18 is reduced after CSE exposure in THP1 cells and monocyte-derived macrophages. Pulmonary cells such as lung epithelial cells or alveolar macrophages could have different responses to cigarette smoke exposure in terms of cellular NLRP3 protein levels, while monocytes or monocyte-derived macrophages have decreased NLRP3 protein levels with cigarette smoke leading to immunosuppression. Indeed, we did not observe significant changes in NLRP3 protein levels in A549 alveolar epithelial cells and Beas-2B bronchial epithelial cells after CSE exposure (data not shown). Another possibility is that increased IL-1β and IL-18 in COPD is mainly derived from a pathway other than NLRP3 inflammasome activation. Although IL-18 knockout mice exhibit reduced pulmonary inflammation and emphysema compared to wild-type mice after cigarette smoke [21, 24], pulmonary inflammation occurred independent of NLRP3/caspase-1 axis after four weeks of cigarette smoke exposure [25]. To clarify these points, further studies are necessary.

Cigarettes smoke is a mixture of more than 4500 chemical compounds [26]. Cigarette smokers inhale and absorb many toxic compounds both in vaporous and particulate phases by burning cigarettes. Immune modulation by cigarette smoke results from the sum of all CSE compounds over time rather than a single compound [27]. We used CSE stored in a vacuum sealed bottle at -80 °C and thereby minimizing the potential confounding effect that might occur by dissipating reactive intermediates. It is unclear to what extent the cigarette smoke in the airway is absorbed into systemic circulation in a human body. However, the dose range of CSE in our study seems appropriate to study immunologic effects based on previous studies [28–30], although the degree of systemic absorption may differ individually [31].

## Conclusions

In summary, our study demonstrates that CSE induces degradation of NLRP3 protein via the ubiquitin proteasome system in human monocytes and macrophages. This CSE-induced degradation is not prevented by LPS, a known stimulus of NLRP3; CSE diminished the formation of NLRP3 inflammasomes and subsequent release of IL-1β and IL-18. Future studies will be carried out to specifically explore molecular targets capable of restoring inflammasome cytokine release and microbial clearance in smokers. This may serve as a unique opportunity to prevent smoking-related morbidity and mortality secondary to infection.

## Abbreviations

CHX: Cycloheximide
COPD: Chronic obstructive pulmonary disease
CSE: Cigarette smoke extract
DAMP: Danger-associated molecular pattern
IL: Interleukin
LPS: Lipopolysaccharide
NLR: NOD-like receptor
NOD: Nucleotide-binding oligomerization
PAMP: Pathogen-associated molecular pattern
SDS-PAGE: Sodium dodecyl sulfate–polyacrylamide gel electrophoresis
TCA: Trichloroacetic acid
